# NudCD1 as a prognostic marker in colorectal cancer and its role in the upregulation of cellular spindle assembly checkpoint genes and LIS1 pathways

**DOI:** 10.1186/s12885-022-10041-4

**Published:** 2022-09-14

**Authors:** Wen-Ming Feng, Hui Gong, Yong-Chun Wang, Yao Wang, Tao Xue, Ting Zhang, Ge Cui

**Affiliations:** 1 Department of Surgery, The First Affiliated Hospital of Huzhou University, No.158 GuangChangHou Road, Huzhou, 313000 China; 2 Central Laboratory, The First Affiliated Hospital of Huzhou University, Huzhou, 313000 China; 3 Department of Pathology, Schools of Medicine and Nursing Science, Huzhou University, Huzhou Central Hospital, Huzhou, 313000 China; 4 Department of Pathology, The First Affiliated Hospital of Huzhou University, Huzhou, 313000 China

**Keywords:** Colorectal cancer, LIS1 pathway, NudCD1, Prognosis marker, Spindle assembly checkpoint genes

## Abstract

**Objective:**

To investigate the role of NudCD1 in spindle assembly checkpoint regulation and in the prognosis of colorectal cancer.

**Methods:**

Immunohistochemical staining was used to detect in situ expression of NudCD1 in 100 colorectal cancer tissue samples. A chi-square test was used to analyse the correlation between the NudCD1 protein expression level of the cancer tissues and clinicopathological features. The Kaplan–Meier survival analysis was used to assess the correlation between the NudCD1 mRNA expression and the three-year survival of patients with colorectal cancer. The impact of NudCD1 on the development of colorectal cancer and the underlying molecular mechanisms were assessed by flow cytometry cell cycle and apoptosis assays after lentiviral overexpression of NudCD1 in two colorectal cancer cell lines. Quantitative real-time PCR was used to assess mRNA expression of the cellular spindle assembly checkpoint genes BUB1, BUBR1, MAD1, CDC20 and MPS1, as well as the downstream genes LIS1, DYNC1H1, and DYNLL1 in the NudC/LIS1/dynein pathway.

**Results:**

Compared with normal intestinal tissue (8.00% with high expression), the expression of NudCD1 protein in colorectal cancer tissue was significantly higher (58.00% with high expression, *P* < 0.01). In addition, expression of NudCD1 significantly correlated with the degree of tumour differentiation and the TNM staging (*P* < 0.01), as well as the depth of invasion of the primary tumour and lymph node metastasis (*P* < 0.05). However, there was no correlation with gender, age, tumour site, gross type, tumour size or distant metastasis. The Kaplan–Meier survival analysis showed that patients with high NudCD1 expression in colorectal cancer tissues had a significantly shorter survival time than those with low expression of NudCD1 (*P* < 0.01). Compared with the transfection of the empty vector, colon cancer HT-29 cells with overexpressed NudCD1 had significantly increased mRNA levels of BUBR1, MPS1 and LIS1. The DNA synthesis phase (S phase) was significantly shorter in cells overexpressing NudCD1 than in the control group (43.83% ± 1.57%, *P* < 0.05), while there was no difference in apoptosis in the two groups.

**Conclusion:**

NudCD1 can serve as a valuable prognostic marker for colorectal cancer. It may be involved in the regulation of spindle-assembly checkpoint-gene expression and the LIS1 pathway of colorectal cancer cells.

**Supplementary Information:**

The online version contains supplementary material available at 10.1186/s12885-022-10041-4.

## Introduction

Colorectal cancer (CRC) is one of the most common malignant tumours in the world [1], and its occurrence and development are related to gene mutation, poor lifestyle choices, environmental changes and other factors [2]. According to statistics, there are about one million new cases of CRC in the world every year, and the mortality rate is about 20.51%. In the case of distant metastasis, the five-year survival rate is only 6% [1]. Therefore, screening for molecular markers that can reflect the early course and prognosis of colorectal cancer is of great significance for inhibiting tumour progression and prolonging survival [3].

It has recently been reported that the nuclear distribution of the gene C (NudC)/lissencephaly 1 (LIS 1)/dynein pathway and its family members is involved in the regulation of mitosis, intracellular transport of material, cell cycle and cell migration. When the function of this pathway becomes disordered, cells may exhibit chromosome aneuploidy and other malignant cytological changes [4]. A previous study has shown that in HeLa cells, silencing of the NudC family member – the NudC domain-containing 3 (NudCD3 or NudCL) gene – induces dynein dislocation and aggregation causing a series of mitotic abnormalities [5]. NudCL overexpression can also lead to mitotic abnormalities, including microtubular structural changes in cell division, formation of multinucleated cells and abnormalities in cytokinesis that ultimately inhibit cell proliferation [6]. Other studies have shown that another NudC family member, the NudC domain-containing 1 (NudCD1), also known as the chronic myelogenous leukaemia tumour antigen 66 (CML66), is overexpressed in a variety of tumour cell lines or tissues [7]. We hypothesised that NudCD1 has functions similar to those demonstrated by NudCL. NudCD1 is a tumour antigen with a wide immunogenic range, located on human chromosome 8q23.3 and is found on screening the serum of patients with chronic myeloid leukaemia using serological analysis of recombinant tumour cDNA expression libraries. In addition to chronic myelogenous leukemia cells, NudCD1 is also overexpressed in lung cancer, melanoma, prostate cancer, pancreatic cancer, colorectal cancer cell lines and tumour tissues, but not in normal human tissues except for the testis and heart tissue [8–10]. Wang et al. [11] silenced NudCD1 in HeLa cells using a short hairpin RNA (shRNA) demonstrating a loss of the ability to proliferate, invade and metastasise, suggesting that NudCD1 is not only a tumour-antigen marker but also an oncogene that directly regulates tumour progression.

In mitosis, the spindle-assembly checkpoint (SAC) controls the proper attachment to and the alignment of chromosomes on the spindle. The SAC detects errors and induces a cell cycle arrest in metaphase, preventing chromatid separation. SAC-related gene mutations can lead to abnormal chromosome segregation resulting in aneuploidy syndrome and atypical changes in cytological morphogenesis [12]. The SAC monitors the attachment of microtubules to chromosomes and ensures proper tension between sister chromatids during mitosis, or homologous chromosomes during meiosis, to ensure the equal distribution of chromosomes into the daughter cells [13]. Recent studies have shown that mutations and deregulation in SAC-related genes, including budding uninhibited by benzimidazole-1 (BUB1) and BUB3 [14], BUB1-related kinase (BUBR1), BUB1B, mitotic arrest-deficient-3 (MAD3) [15], the monopolar spindle 1 (Mps1/TTK) [16], MAD1 [17] and cell division cycle-20 (CDC20) [18] are related to the occurrence and development of CRC. Mutations in the LIS1 gene are responsible for human type I lissencephaly. LIS 1 co-localizes with dynein in the kinetochore, centrosome and anterior membrane of the chromosome and can regulate dynein-mediated cell mitosis and cell migration [19].

We used immunohistochemistry (IHC) to detect in situ NudCD1 expression in paraffin-embedded colorectal cancer tissue and studied the correlation between NudCD1 expression, clinicopathological features and prognosis in colorectal cancer patients. The aim was to explore the possibility of using NudCD1 as a diagnostic and prognostic marker in colorectal cancer. We studied colon-cancer cell lines with overexpressed NudCD1 genes to assess changes in the cell cycle, apoptosis, SAC-related gene expression and the LIS1 pathway to explore the possible mechanism of NudCD1 involvement in cell mitosis and abnormal chromosome segregation in colorectal cancer.

## Methods

### Sampling

Clinical samples were obtained from 100 patients with colorectal cancer who had not received any anti-tumour therapies prior to sample collection. Resected colorectal cancer tissue in wax blocks was collected from our hospital for study purposes after diagnosis by the pathologist. The study protocol was reviewed and approved by the medical ethics committee and informed consent was obtained from the patients.

### Follow-up registration, clinicopathological features and prognosis of colorectal cancer

Clinicopathological data of enrolled patients with colorectal cancer were collected, including gender, age, tumour location, gross type, tumour size, neurovascular involvement, differentiation, primary tumour invasion, lymph node metastasis and distant metastasis. Pathological patterns and differentiation rankings of tumours were defined according to the 2010 World Health Organization diagnostic criteria for gastrointestinal tumours [8]. The tumour, nodes, metastasis (TNM) staging was done as per the 2009 American Joint Committee on Cancer and the International Union Against Cancer TNM staging system for colorectal cancer (7^th^ edition) [20]. The survival status of patients included in the study was assessed by follow-up telephonic consultations and household surveys by the public security systems. To record the current living status and to calculate the three-year survival time in months, we used the following codes: death = 0, survival = 1, and lost to follow-up = 2.

### Immunohistochemical staining to detect in situ NudCD1 expression in tissue samples

The paraffin tissue was sectioned, baked at 65 °C overnight, dewaxed in xylene, washed with alcohol and water and then soaked in 3% H_2_O_2_ solution for 15 min in the dark. The tissue was then immersed in 0.01 mol/L citrate buffer (pH 6.0) for three minutes in a pressure cooker for antigen retrieval. Next, it was cooled with tap water, washed with phosphate buffer saline (PBS) and incubated with the NudCD1 primary antibody (1:200, ab126902, abcam) at 4 °C overnight. Langerhans cells of human skin were used as a positive control, and a sample stained with PBS in place of the primary antibody was used as a negative control.

The following day, the sectioned piece was washed with PBS and incubated with goat anti-rabbit HRP IgG secondary antibody at room temperature for 15 min. Next, it was washed with PBS and incubated with 3,3’-diaminobenzidine (DAB) solution for a length of time appropriate for colouration under a microscope. The sample was then stained with Harris haematoxylin for two minutes, differentiated with hydrochloric acid in ethanol and dehydrated with gradient alcohol. Next, it was made transparent with xylene, mounted on slides with soluble resin and examined under a microscope.

Positive staining was defined by the presence of brown or tan particles in the cytoplasm of parenchymal cells. Five fields (200X) in each section were examined. Scoring based on the percentage of positive cells was as follows: 0 ~ 5% positive cells – 0 points, 5% ~ 25% – 1 point, 26% ~ 75% – 2 points, and 76% ~ 100% – 3 points. According to the IHC staining intensity, the scoring was as follows: colourless – 0 points, yellowish – 1 point, brownish – 2 points, and tan – 3 points. Stained tissues were read and scored by two pathologists who were blinded to each other’s results, take the average value of the results read by the two doctors as the final score. Tissue staining was scored according to staining intensity and the percentage of positive cells, with 0–4 representing low expression and 5–9 representing high expression. A chi-square test was used to compare differences between the groups.

### Cell culture

Colon cancer cell lines (LoVo, SW620, HCT116, HT-29) were cultured in an L-Glutamine Dulbecco’s Modified Eagle Medium (L-DMEM) and incubated at 37 °C with 5% CO_2_ and saturated humidity in a cell culture incubator (Forma, USA). All cell lines were purchased from Nanjing Cobioer Bioscience Co. Ltd. The cells were digested with 0.25% trypsin for passage, and logarithmic growth-phase cells were collected for the assays described below.

### Real time quantitative polymerase chain reaction to detect NudCD1 expression in different cancer cell lines

The colon cancer cell lines (LoVo, SW620, HCT116, HT-29) were lysed withTRIzol Reagent (Invitrogen Corp., CA, USA) at room temperature for five minutes. Then one-fifth the volume of chloroform (Sinopharm, Beijing, China) was added with oscillation for 15 s. When the solution emulsified completely, it was allowed to sit at room temperature for five minutes and then centrifuged at 12,000xg for 15 min at 4 °C. The supernatant was transferred into another RNase-free Eppendorf (EP) PCR tube with an equal volume of isopropanol (Sinopharm, Beijing, China) and mixed thoroughly. MonScript™ RTIII All-In-One Mix (Monad Biotech Co. Ltd., Wuhan, China) was used for synthesis of cDNA. This was then mixed with ds DNase (Monad Biotech Co. Ltd., Wuhan, China) according to the manufacturer’s protocol. MonAmp™ ChemoHS qPCR Mix (Monad Biotech Co. Ltd., Wuhan, China) was used for performing real time quantitative polymerase chain reaction (qPCR). Samples were incubated at 50 °C for 15 min, followed by 95 °C for 5 min, followed by 32 PCR cycles with the following temperature profile: 95 °C for 15 s, 60 °C for 30 s and 72 °C for 1 min. The primers were as follows: NudCD1-forward, 5’-CCTGCTTCTGTTTGCGCCATG-3; NudCD1 -reverse, 5’-GAAGGCACTCACAAAGGGCTG -3’; GAPDH-forward, 5’-ACAACTTTGGTATCGTGGAAGG-3; and GAPDH-reverse and 5’-GCCATCACGCCACAGTTTC-3’. The relative expression of the corresponding gene mRNA was analysed and expressed as 2 ^−ΔΔCt^ (△ threshold cycle [CT] is the CT value of the target gene in the same sample minus the internal reference CT value). The relative mRNA expression was standardised to the expression level of glyceraldehyde-3-phosphate dehydrogenase (GAPDH) mRNA.

### NudCD1 overexpression in colon cancer cell lines

**Fig. 1:**
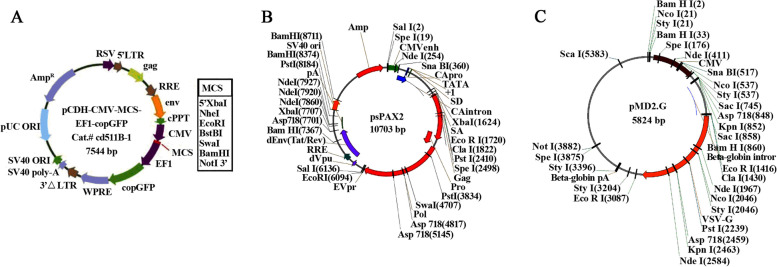
Plasmid profile of NudCD1 overexpressing lentiviral vector system. Plasmid profile of NudCD1 overexpressing lentiviral vector system was structured, including pCDH-GFP vector map (**A**), psPAX2 vector map (**B**) and pMD2.G vector map (**C**)

Colon cancer cell lines were screened by qPCR for NudCD1 expression and divided into two groups for either empty vector control or NudCD1 overexpression (NudCD1 +). A vector for the overexpression of NudCD1 in the colon cancer cell lines was established as follows. The human source vitronectin precursor (VTN) sequence was obtained from the National Center for Biotechnology Information database (Accession No. BC000967) and primers were designed in accordance with the coding DNA sequence (CDS): VTN-F 5’-TGCTCTAGAGCCACCATGGAGGTGGCGGCTAATTG-3’ and VTN-R 5’-CCGGAATTCTTAATTCTCTGTATTTACTTTTATTAAA-3’. Total RNA was extracted by using the TRIzol method, and a reverse transcription kit was used to reverse-transcribe total RNA into cDNA. The plasmid profile of the NudCD1 overexpressing lentiviral vector system is shown in Fig. 1.

Plasmid was extracted for sequencing with the following sequencing primer: CACGCTGTTTTGACCTCCATAGAA. Transfection appeared when the cell fusion degree was up to 90%–95%. Fluorescence expression was observed after four days. Polybrene was then added to a final concentration of 6 µg/mL. If the infection rate was over 90%, amplification was performed.

### The determination of the cycle of colon cancer cells

Cells in the logarithmic phase in each group were collected, resuspended in PBS and counted, after which 1 × 10^5^ cells were transferred to a centrifuge tube and centrifuged at 1000 rpm for five minutes. The supernatant was discarded, and the cell sediment was washed with PBS. Next, 0.5 ml PBS was left in the centrifuge tube, and 5 ml of pre-cooled 75% ethanol was added, fixed and placed for 24 h at -20 °C. The ethanol was discarded after centrifugation and the cell sediment was again washed with PBS. One mL PBS was left in the centrifuge tube and the cells resuspended. This was followed by the addition of 5 µL RNase (10 mg/mL) and incubation at 37 °C for one hour. Next, the sample was stained with 5 μl propidium iodide (PI) (10 mg/mL, Sigma, USA) for 30 min at room temperature with the aim of avoiding light staining. The sample was then examined for cell cycle assay by flow cytometry (BD FACSCalibur, BD Biosciences, CA, USA).

### Observation of apoptosis of the colon cancer cells

The cells in the logarithmic phase in each group were collected, resuspended in PBS and counted, after which 1 × 10^5^ cells were transferred to a centrifuge tube and centrifuged at 1000 rpm for five minutes. The supernatant was discarded, and the cells were gently resuspended with 195 μL Annexin V-FITC binding solution and gently mixed with 5 μl Annexin V-FITC (Annexin V-FITC/PI Apoptosis Detection Kit, Gibco, USA). After incubation in the dark at room temperature for ten minutes, the mixture was centrifuged at 1000 rpm for five minutes, the supernatant was discarded, and the cells gently resuspended with 190 μL Annexin V-FITC binding buffer. Then, 10 μl PI staining solution was added, mixed gently and placed in an ice bath in the dark. Apoptosis was measured by flow cytometry, in which Annexin V-FITC presented green fluorescence, while PI presented red fluorescence.

### Detection of mRNA expression of SAC-related genes in colon cancer cell lines by qPCR

Real-time quantitative PCR was used to detect the mRNA levels of SAC-related genes, including BUB1, BUBR1, MAD1, CDC20 and MPS1/TTK, as well as downstream molecules in the LIS 1/dynein pathway, namely, LIS 1, DYNC1H1 and DYNLL1. The qPCR conditions were the same as the detection method for NudCD1 expression described earlier. Primer sequences and synthesis conditions (following the same detection procedures as in subsection 2.5) are shown in Table 1. The relative mRNA expression was standardised to the expression level of GAPDH mRNA.

**Table 1:** Primer sequence and synthesis condition

GENE	Primer	Length (bp)	Tm (degrees centrigade)
GAPDH	ACAACTTTGGTATCGTGGAAGG	101	60.7
	GCCATCACGCCACAGTTTC		60.54
BUB1	GGAGAAGGAGCCTTTGCCCAG	117	60.14
	GTAGAATTCCCAGGGGTTGGCAG		60.62
BUBR1	CTTCTCCCTACCAGGTAGACCTG	173	60.77
	GGCATTCAGAATCCGCACAAAG		60.75
MAD1	GAGTCTGCCATCGTCCAAGGAG	157	60.03
	CGTGGTGATGTCGATCTGGTAGC		60.04
CDC20	CTCAGGCCATGGCTTTGCACAG	158	60.99
	TCAGGGTCTCATCTGCTGCTGC		61.65
TTK	GGGACCCAAAACAGAGGATATCC	134	60.8
	ACCAACAAGTTGGCCCAGAAC		60.82
LIS1	TCTTGCTGTCTGGATCCAGAGAC	156	60.8
	GGGTCTTGTCATCAGCACAACTC		60.82
DYNC1H1	TGGAAGTCAACGTCACCACCTC	128	60.8
	GGTTGAGATGGCATTGGACAGTG		60.82
DYNLL1	CTCAGGCGCTGGAGAAATACAAC	95	60.8
	GATGCAATGCCAGGTGGGATTG		60.82

### Statistical analysis

The statistical package IBM SPSS Statistics for Windows, Version 19.0. (Armonk, NY) was used for data analysis. All data were expressed as mean ± standard deviation. Measurement data were compared using t-tests, and the count data were compared using the chi-square test. The Kaplan–Meier survival analysis was used for univariate survival analysis of colorectal cancer patients. The log-rank test was used to draw the survival curve. A *P* value < 0.05 was considered statistically significant.

## Results

### The in situ expression of NudCD1 in colorectal cancer tissues

**Fig. 2:**
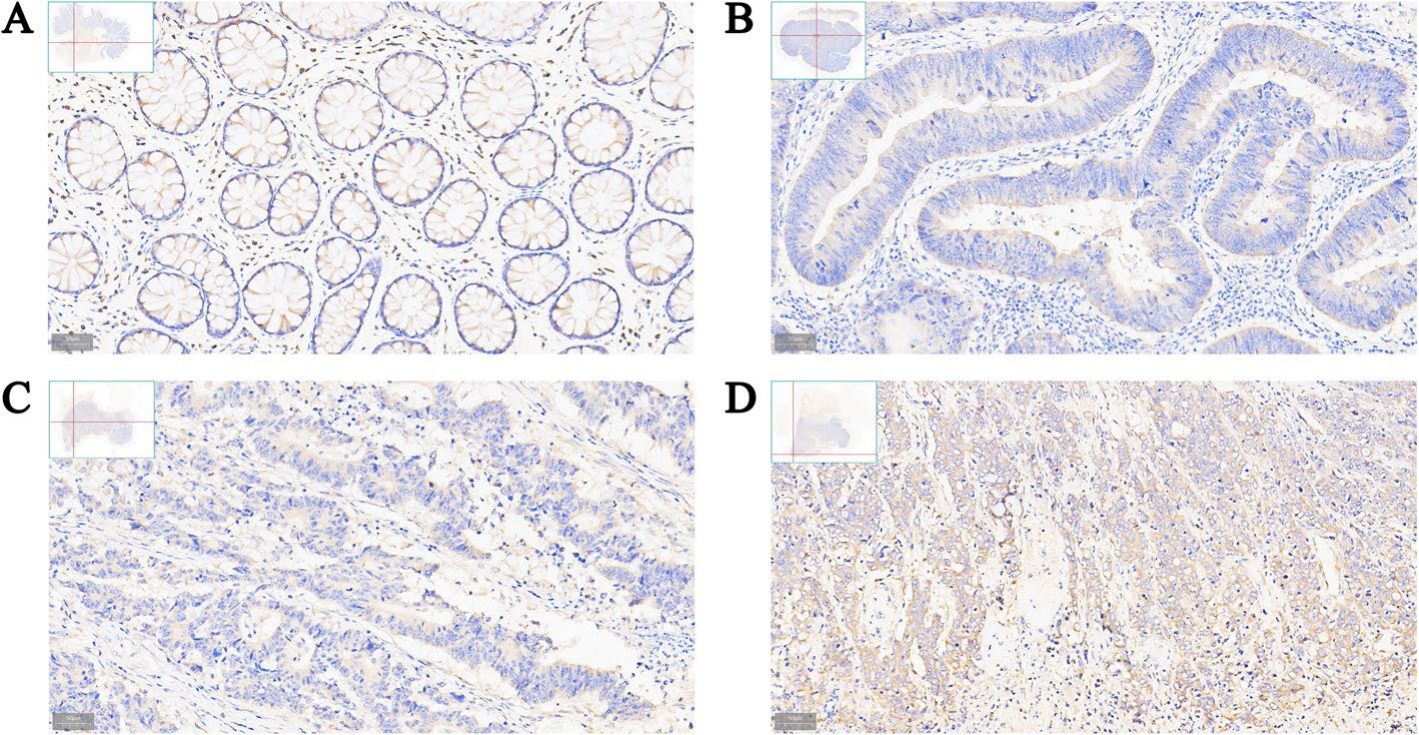
Expression of NudCD1 protein in normal tissue and colorectal tumor tissue. NudCD1 was mainly expressed in the cytoplasm of intestinal mucosal gland, with brown or tan particles of different area and brightness. Compared with normal intestinal tissues (**A** high expression of 8.00%, staining score was 5.2 ± 0.4), protein expression of NudCD1 was significantly higher in colorectal cancer tissue with different differentiation (**B** Well-differentiated, high expression of 58.00%, staining score was 8.2 ± 0.8, χ2 = 56.536, *P* < 0.01; **C**. Moderately differentiated, high expression of 62.30%, staining score was 9.7 ± 0.7, χ2 = 59.752, *P* < 0.01; **D**. Poorly differentiated, high expression of 68.40%, staining score was 9.9 ± 0.6, χ2 = 66.658, *P* < 0.01). Images were taken at magnification of 200 ×

IHC staining was used to detect in situ expression of NudCD1 in colorectal cancer tissues. The results showed that NudCD1 was mainly expressed in the cytoplasm of the intestinal mucosal glands, with brown or tan particles of different area and brightness. Compared with normal intestinal tissues (Fig. 2A: with high expression of 8.00% and staining score of 5.2 ± 0.4), protein expression of NudCD1 was significantly higher in colorectal cancer tissue. Moreover, it was found that the protein expression of NudCD1 was significantly higher in poorly differentiated colorectal cancer as compared with moderately differentiated and well differentiated colorectal cancer (Fig. 2B: well differentiated, with high expression of 58.00% and staining score of 8.2 ± 0.8, χ2 = 56.536, *P* < 0.01; Fig. 2C: moderately differentiated, with high expression of 62.30% and staining score of 9.3 ± 0.7, χ2 = 59.752, *P* < 0.01; Fig. 2D: poorly differentiated, with high expression of 68.40% and staining score of 9.9 ± 0.6, χ2 = 66.658, *P* < 0.01).

### Correlation of in situ NudCD1 expression in colorectal cancer tissues with clinicopathological features

The chi-square test was used to assess the correlation between NudCD1 protein expression and clinicopathological features in colorectal cancer. Results showed that NudCD1 protein expression in colorectal cancer tissue correlated significantly with tumour differentiation and TNM staging (*P* < 0.01), as well as with invasion by the original tumour and lymph node metastasis (*P* < 0.05). However, there was no significant correlation with gender, age, tumour site, gross type, tumour size, neurovascular involvement or distant metastasis (*P* > 0.05, Table 2).

**Table 2:** The correlation between NudCD1 protein in colorectal cancer tissues with clinicopathological features

**Clinicopathological features**	*n* = *100*	NudCD1 protein level ( *n)*		χ2	*P*
		high expression(58)	low expression(42)		
**Sex**					
Male	67	37	30	0.642	0.423
Female	33	21	12		
**Age (Years)**					
< 60	22	9	13	3.382	0.066
≥ 60	78	49	29		
**Tumor location**					
Ascending colon( including Cecum)	35	21	14	1.697	0.791
Transverse colon	8	6	2		
Descending colon	13	8	5		
Sigmoid colon	11	6	5		
Rectum	33	17	16		
**Gross type**					
Polyp	21	9	12	3.877	0.144
Invasive	38	21	17		
Ulcerative	41	28	13		
**Tumor diameter (cm)**					
< 3	42	20	22	3.203	0.073
≥ 3	58	38	20		
**Neurovascular invasion**					
No invasion	26	11	15	2.673	0.102
Neuro/vascular invasion	74	45	29		
**Differentiation**					
High	32	14	18	10.881	0.004
Medium	25	11	14		
Low	43	33	10		
**Primary tumor invasion**					
T1, T2	31	13	18	4.760	0.029
T3, T4	69	45	24		
**Lymph metastasis**					
N0, N1	72	37	35	4.614	0.032
N2, N3	28	21	7		
**Distant metastasis**					
M0	91	51	40	1.588	0.208
M1	9	7	2		
**TNM classification**					
I, II	63	27	36	13.176	0.000
III, IV	39	31	8		

### The correlation of in situ NudCD1 expression in colorectal cancer tissues in patients with an average survival of three years

**Fig. 3:**
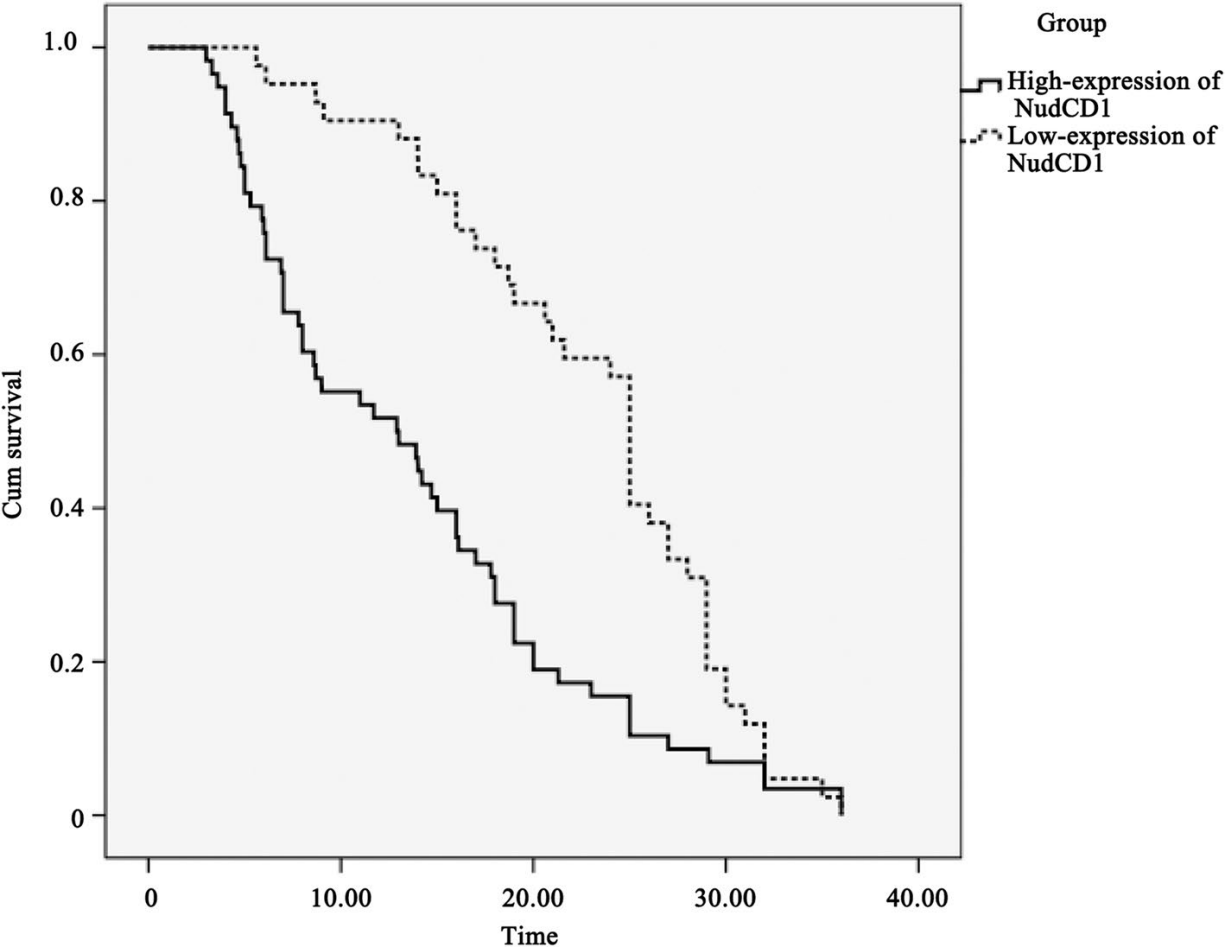
The Kaplan–Meier survival curves of patients with colorectal cancer according to different NudCD1 protein expressions. Kaplan–Meier survival analysis showed that survival time of patients with high NudCD1 expression in colorectal cancer tissues was significantly shorter than those with low NudCD1 expression

The Kaplan–Meier survival analysis showed that in colorectal cancer patients with high NudCD1 protein expression, the three-year mean survival time was 13.8 ± 1.2 months with a median survival time of 12.9 ± 3.2 months, while in patients with low NudCD1 expression it was 22.9 ± 1.2 months with a median survival time of 25.0 ± 0.5 months (see Table 3). The Kaplan–Meier survival analysis curve is shown in Fig. 3. The log-rank (Mantel–Cox) test showed that the survival time of patients with high NudCD1 expression in colorectal cancer tissue was significantly shorter than those with low NudCD1 expression (χ2 = 13.793, *P* < 0.01, Table 4).

**Table 3:** Patient survival after colorectal cancer diagnosis according to NudCD1 protein expressions

Group	Mean				Median			
	Estimate	Std. Error	95% Confidence Interval		Estimate	Std. Error	95% Confidence Interval	
			Lower Bound	Upper Bound			Lower Bound	Upper Bound
High-expression of NudCD1	13.8	1.2	11.5	16.1	12.9	3.2	6.7	19.1
Low-expression of NudCD1	22.9	1.2	20.6	25.3	25.0	0.5	24.1	25.9
Overall	17.6	1.0	15.8	19.5	17.0	1.5	14.0	20.0

**Table 4:** Overall Comparisons of Survival Time of different expression of NudCD1 protein in colorectal cancer tissue

	Chi-Square	df	Sig
Log Rank (Mantel-Cox)	13.793	1	.000
Breslow (Generalized Wilcoxon)	22.743	1	.000
Tarone-Ware	20.282	1	.000

### The relative expression level of NudCD1 in colorectal cancer cell lines using qPCR

**Fig. 4:**
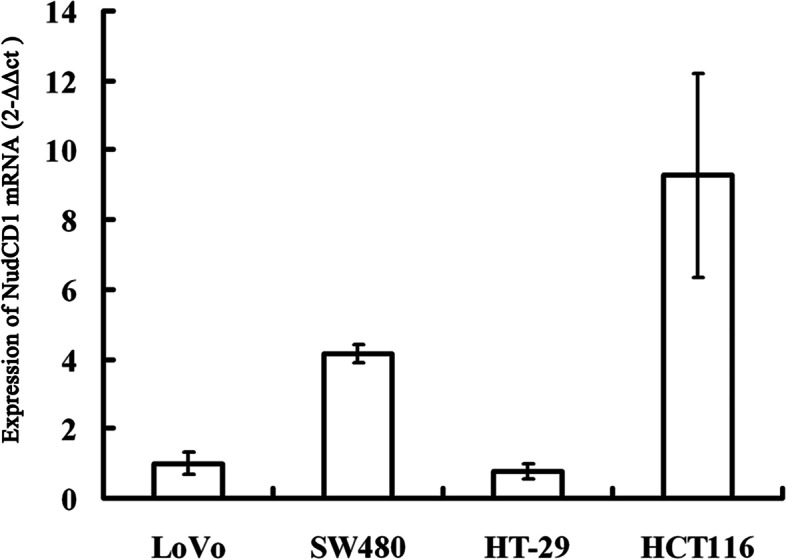
Expression of NudCD1 mRNA in colorectal cell lines. qPCR showed that HT-29 and HCT116 cells had the minimum and maximum levels of NudCD1 mRNA expression, respectively

A quantitative PCR was used to detect the NudCD1 expression level in CRC cell lines, including LoVo, SW620, HCT116 and HT-29. Results showed that HT-29 and HCT116 cells had the minimum (2^−ΔΔCt^ = 0.779 ± 0.261) and maximum (2^−ΔΔCt^ = 9.278 ± 0.116) levels of NudCD1 expression, respectively. HT-29 cells were used as the NudCD1 gene overexpression cell line (Fig. 4).

### Construction of the NudCD1 overexpression plasmid and validation

**Fig. 5:**
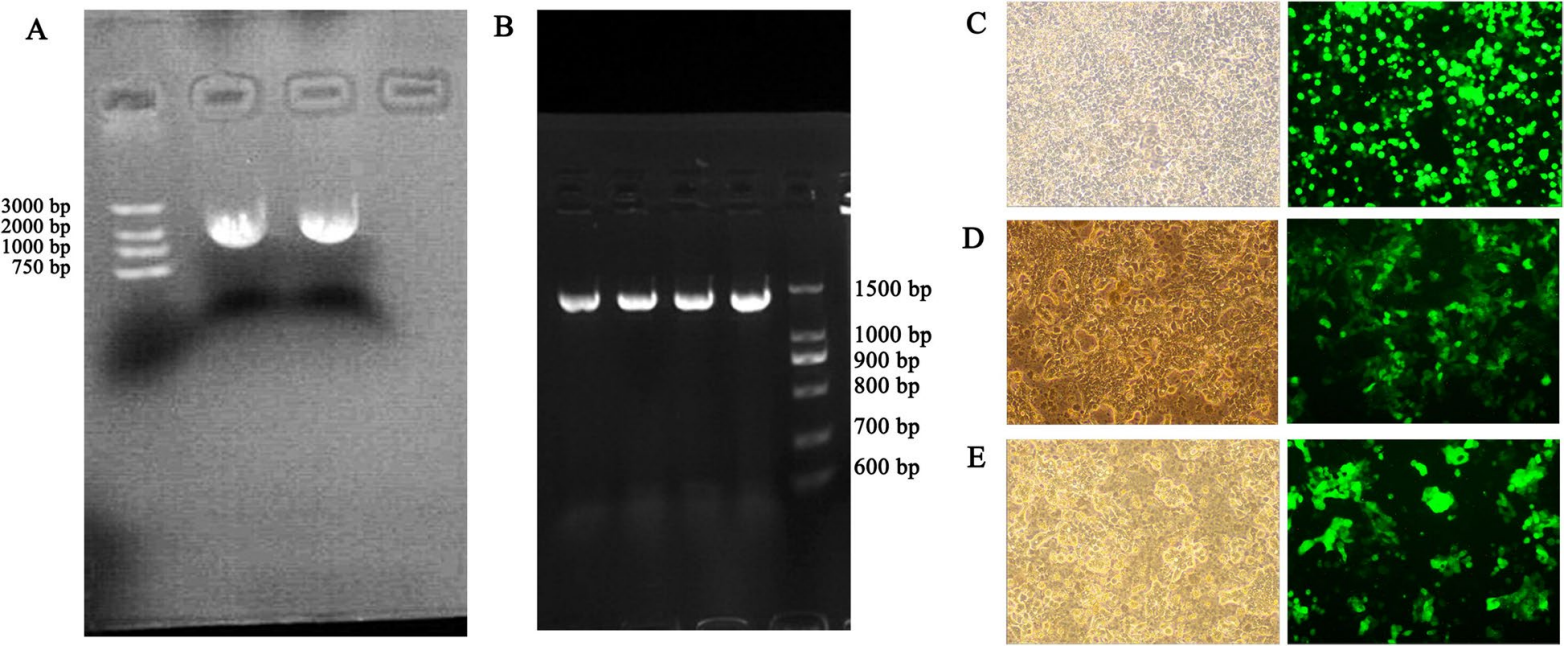
Construction of NudCD1 overexpression plasmid and validation. cDNA NudCD1 primer PCR electrophoresis, the size of PCR products were consistent with the open reading frame (ORF) of NudCD1(1752 bp) (**A**). NudCD1 colony PCR products, four lanes were positive (**B**). PCR positive colonies were verified by sequencing extracted plasmid. 293 T cells infected with lentivirus plasmid for 48 h (**C**), lentivirus was observed by fluorescence microscopy 48 h after transfection, indicating that lentiviral production was in good condition. NudCD1 overexpressed lentivirus infected colon cancer cells HT-29 (bright-field and fluorescence) (**D**). Empty viral vector infected colon cancer cells HT-29 (bright-field and fluorescence) (**E**)

The size of the cDNA NudCD1 primer PCR products was consistent with the open reading frame of NudCD1 (1752 bp) via electrophoresis (Fig. 5A). Four lanes of the NudCD1 colony PCR products were positive (Fig. 5B). PCR-positive colonies were verified by sequencing extracted plasmid. Sequencing results (see supplemental Fig. 1) were consistent among the four, and plasmid construction was completed. In the 293 T cells infected with lentivirus plasmid for 48 h, lentivirus was observed by fluorescence microscopy 48 h after transfection (Fig. 5C), NudCD1 overexpressed lentivirus (Fig. 5D), and empty viral vector (Fig. 5E) infected colon cancer cells HT-29 (bright-field and fluorescence) indicating that lentiviral production was in good condition.

### mRNA expression of SAC-related genes in colon cancer cell lines

**Fig. 6:**
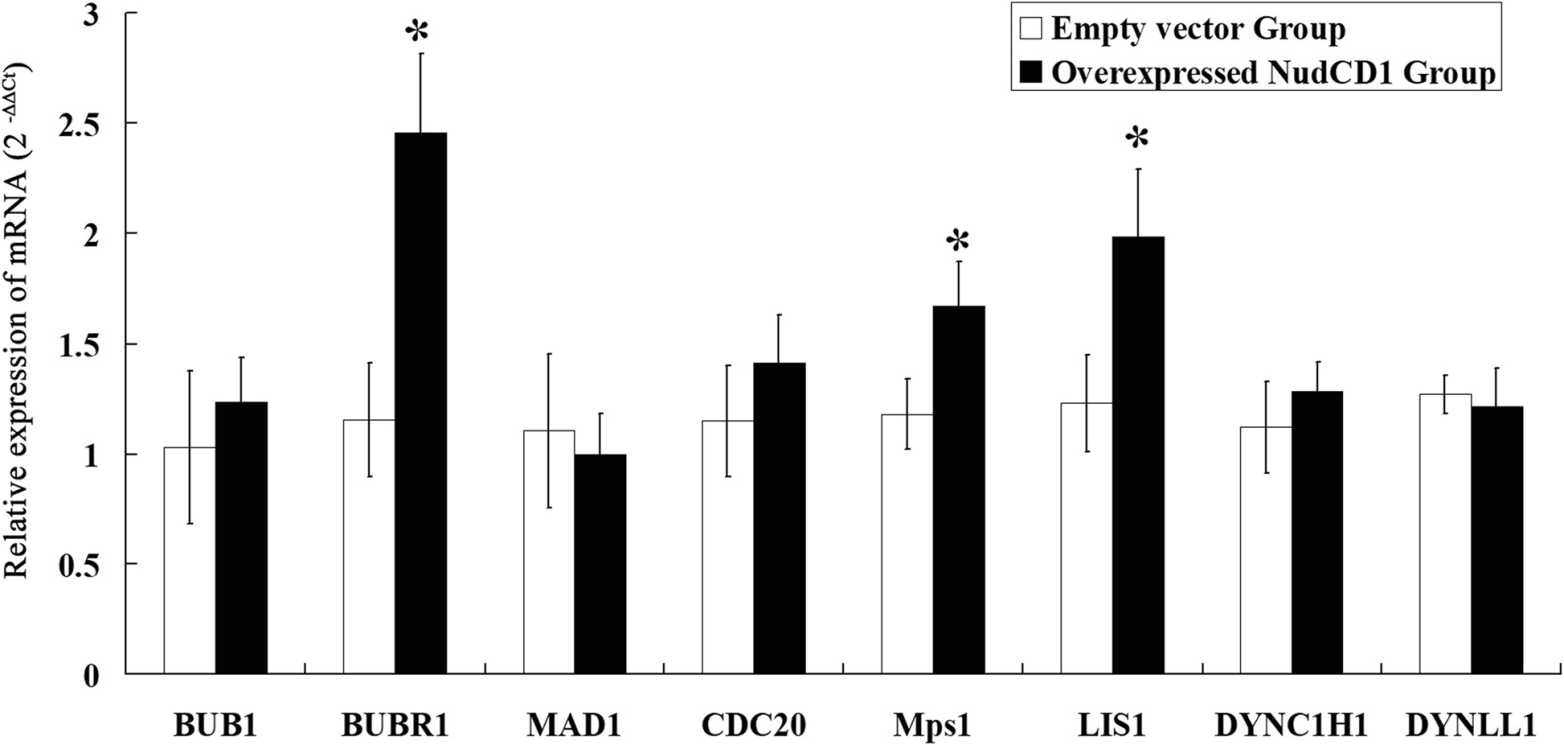
Detection of mRNA expression of SAC-related genes in colon cancer cell lines. Compared to the transfection of the empty vector, colon cancer cells with overexpressed NudCD1 had significantly increased mRNA levels of BUBR1, MPS1 and LIS1, while BUB1, MAD1, CDC20, DYNC1H1and DYNLL1in HT-29 cells had no significant difference (*P* < 0.05)

Compared with the transfection of the empty vector, colon cancer HT-29 cells with overexpressed NudCD1 had significantly increased mRNA levels of BUBR1 (1.154 ± 0.258 *vs* 2.455 ± 0.359, *P* < 0.05), MPS1 (1.179 ± 0.158 *vs* 1.671 ± 0.200, *P* < 0.05) and LIS1 (1.228 ± 0.220 *vs* 1.97 ± 0.304, *P* < 0.05). However, BUB1 (1.028 ± 0.347 *vs* 1.234 ± 0.201, *P* > 0.05), MAD1 (1.105 ± 0.348 *vs* 0.996 ± 0.185, *P* > 0.05), CDC20 (1.149 ± 0.252 *vs* 1.412 ± 0.216, *P* > 0.05), DYNC1H1 (1.121 ± 0.208 *vs* 1.284 ± 0.134, *P* > 0.05) and DYNLL1 (1.270 ± 0.085 *vs* 1.215 ± 0.172, *P* > 0.05) in HT-29 cells showed no significant difference (Fig. 6).

### The cell cycle and apoptosis of colon cancer cells with different NudCD1 expression levels

**Fig. 7:**
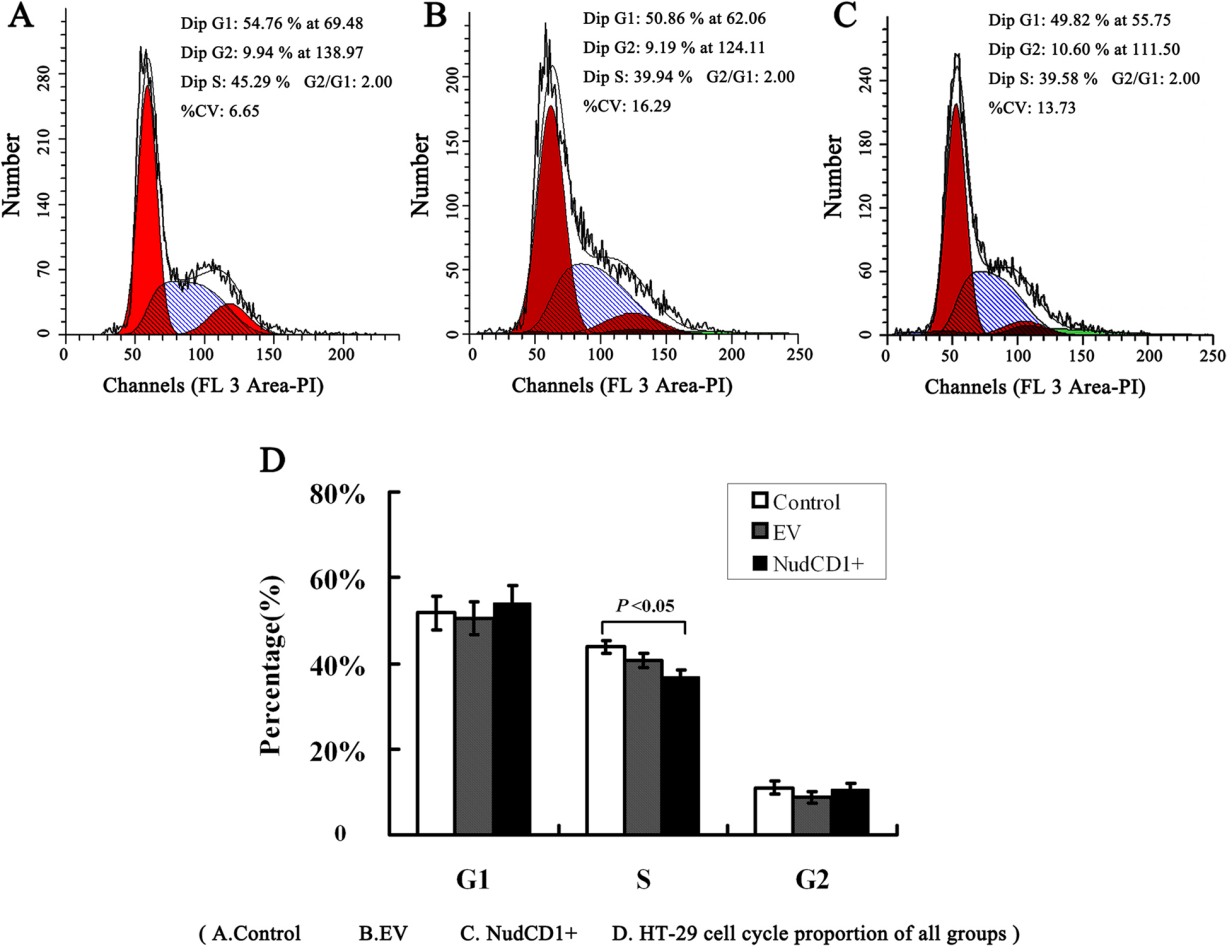
The cell cycle of colon cancer cells with different NudCD1 expressions. Colon cancer cells were stained with propidium iodide (PI) and analyzed with flow cytometer. Flow cytometry showed that the length of S phase in the NudCD1 overexpression colon cancer cells (36.96% ± 2.27%) was significantly shorter than in HT-29 cells without transfection (43.83% ± 1.57%, *P* < 0.05)

Flow cytometry showed that the length of the DNA synthesis phase (S phase) in the NudCD1 overexpression colon cancer cells (36.96% ± 2.27%) was significantly shorter than in colon cancer cells without transfection (43.83% ± 1.57%, *P* < 0.05), while colon cancer cells transfected with empty vector (40.74% ± 0.79%) showed no significant difference from colon cancer cells without transfection (*P* > 0.05). The G1 and G2 phases in the NudCD1 overexpression colon cancer cells showed no significant difference compared with those without transfection or those transfected with empty vector (*P* > 0.05, Fig. 7).

**Fig. 8:**
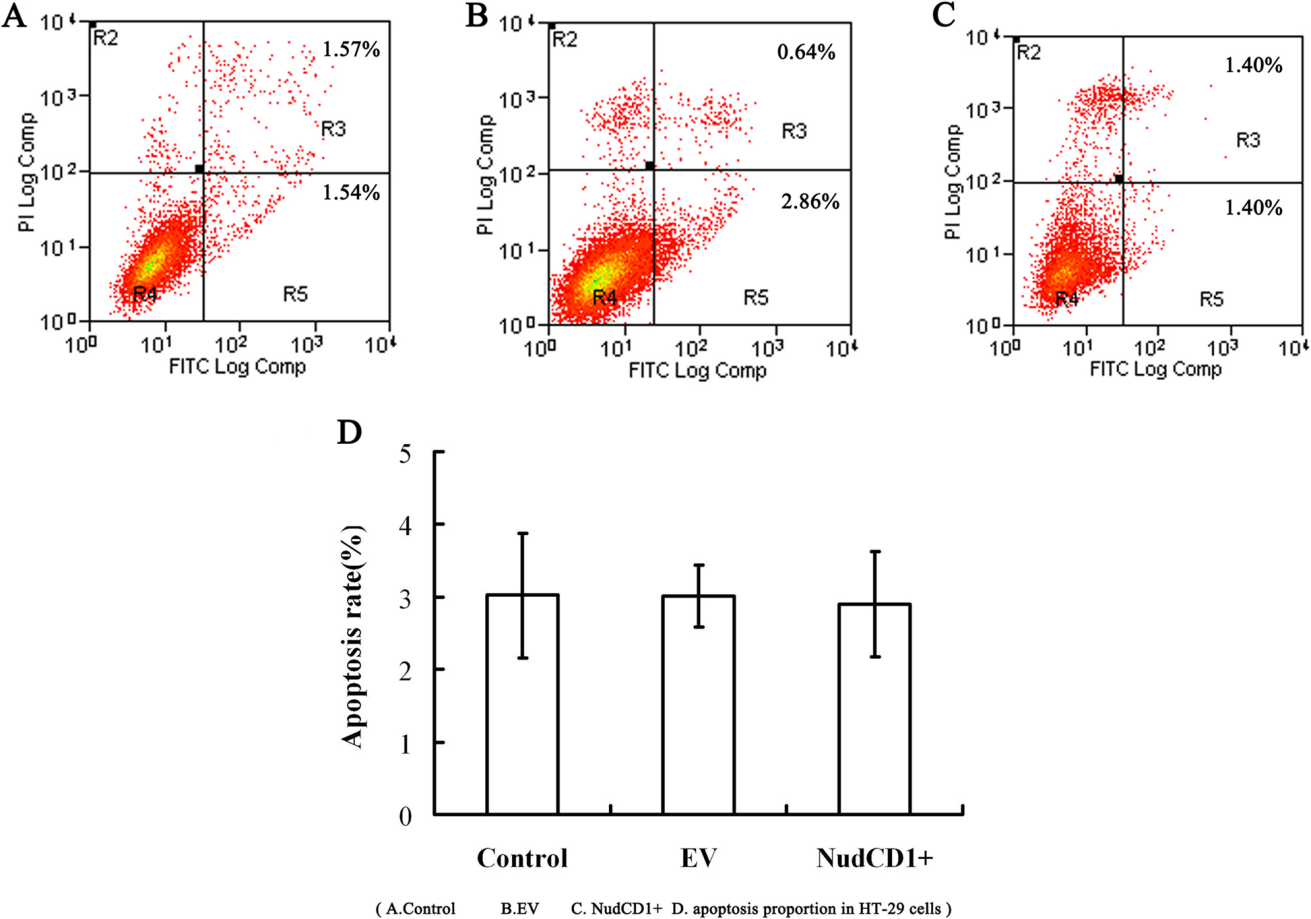
The apoptosis of colon cancer cells with different NudCD1 expressions. Colon cancer cells were double-stained with Annexin V-FITC/PI Apoptosis Detection Kit and analyzed with flow cytometer. The NudCD1 overexpression colon cancer cells had an apoptosis rate of 2.91% ± 0.73%, which is not significantly different from those without transfection (3.01% ± 0.42%) or transfected with empty vector (3.02% ± 0.86%, *P* > 0.05)

The NudCD1 overexpression colon cancer cells had an apoptosis rate of 2.91% ± 0.73%, which was not significantly different from those without transfection (3.01% ± 0.42%) or those transfected with empty vector (3.02% ± 0.86%, *P* > 0.05, Fig. 8). This study showed that NudCD1 had no significant effect on apoptosis of colon cancer cells.

## Discussion

CRC is a common malignant tumour. According to the World Health Organisation, the number of CRC patients in the world is projected to increase by 77% by 2030, and the number of deaths due to CRC by 80% [21]. Colon cancer differs in its pathogenesis, symptoms, tumour pathology and molecular biology due to different local micro-environments, histomorphology, physiology and metabolic characteristics [22]. Therefore, it is urgent to screen for early markers.

This is the first study to verify that the expression of NudCD1 protein in colorectal cancer is significantly higher than that in normal intestinal mucosa, and that its degree of expression is related to the degree of differentiation of the tumour, the TNM stage, the depth of invasion of the primary tumour and lymph node metastasis. This study also found that the survival time of colorectal cancer patients with high expression of NudCD1 protein was significantly shorter than that of patients with low expression of NudCD1 protein. These results suggest that NudCD1 may be a valuable marker for evaluating the course and prognosis of colorectal cancer.

In this study, the expression of NudCD1 in colorectal cancer tissue and adjacent normal mucosa was determined by IHC, and the results showed that NudCD1 protein expression was significantly higher (*P* < 0.01) in colorectal cancer tissues, while normal intestinal tissues had weak or no expression. NudCD1 protein expression in colorectal cancer tissues had a remarkable correlation with tumour differentiation and TNM staging (*P* < 0.01). It also showed some correlation with primary tumour invasion and lymph node metastasis (*P* < 0.05). The Kaplan–Meier survival analysis showed that patients with high NudCD1 expression in colorectal cancer tissues had a significantly shorter survival time than those with low NudCD1 expression (*P* < 0.01). This study showed that the NudCD1 expression level is consistent with the progress of colorectal cancer.

Quantitative PCR assay was adopted to detect the mRNA levels of relevant molecules in colon cancer HT-29 cells with differing levels of NudCD1 expression. The results showed that NudCD1 overexpressing colon cancer HT-29 cells had significantly higher mRNA expressions of BUBR1, MPS1 and LIS1, indicating that NudCD1 promotes the occurrence of colorectal cancer by up-regulating SAC-related genes and the LIS1 pathway.

It has been reported that active cell cycle and apoptosis signals play a key role in tumour development [23, 24]. The cell cycle progression and apoptosis of colon cancer cells with differing levels of NudCD1 expression were examined by flow cytometry. Compared with the untreated control group, the S phase was shortened in NudCD1 overexpressing cancer cells, indicating that NudCD1 overexpression interfered with DNA replication of colon cancer cells, potentially exacerbating the formation of atypical cancer cells.

Additionally, we found that the expression of the spindle checkpoint genes BUBR1 and MPS1 was significantly increased. The mRNA expression of LIS1, a downstream molecule of NUDC / LIS1 / dynein pathway, was also significantly increased in NudCD1 overexpressed colon cancer cells, whereas the S phase was relatively shortened. Mutations of the spindle checkpoint gene can lead to abnormal chromosome segregation [3] and disorder of the regulation function of the NUDC / LIS1 / dynein pathway leading to malignant cytological changes such as chromosome aneuploidy [22]. The shortening of the S phase, insufficient time for DNA and histone synthesis and the interference of normal chromosome replication and cell cycle lay the cell biology foundation for cell carcinogenesis. These results suggest that NudCD1 may be involved in the development of colorectal cancer by regulating spindle checkpoint gene expression and the LIS1 pathway.

## Conclusion

Colorectal cancer is a common malignant tumour occurring in the gastrointestinal tract. It has a high incidence rate and mortality rate. This study found that the expression of NudCD1 protein in colorectal cancer tissues was significantly higher than that in normal intestinal tissue, and its expression level was consistent with the progression of colorectal cancer. NudCD1 may be an important marker for the prognosis of colorectal cancer. The potential mechanisms at a subcellular level may be explained by the upregulation of the cellular spindle-assembly checkpoint genes and LIS1 pathways. These results suggest that NudCD1 may be involved in regulating spindle assembly checkpoint gene expression and the LIS1 pathway of colorectal cancer cells.

## Supplementary Information

**Additional file 1.** 

## Data Availability

The datasets used and analyzed during the current study are avaliable from the corresponding author on reasonable request.
