# Supplementary figures and images for: NudCD1 as a prognostic marker in colorectal cancer and its role in the upregulation of cellular spindle assembly checkpoint genes and LIS1 pathways
Source: BMC Cancer. 2022 Sep 14;22:981. doi: 10.1186/s12885-022-10041-4 (PMC9476325; doi:10.1186/s12885-022-10041-4)

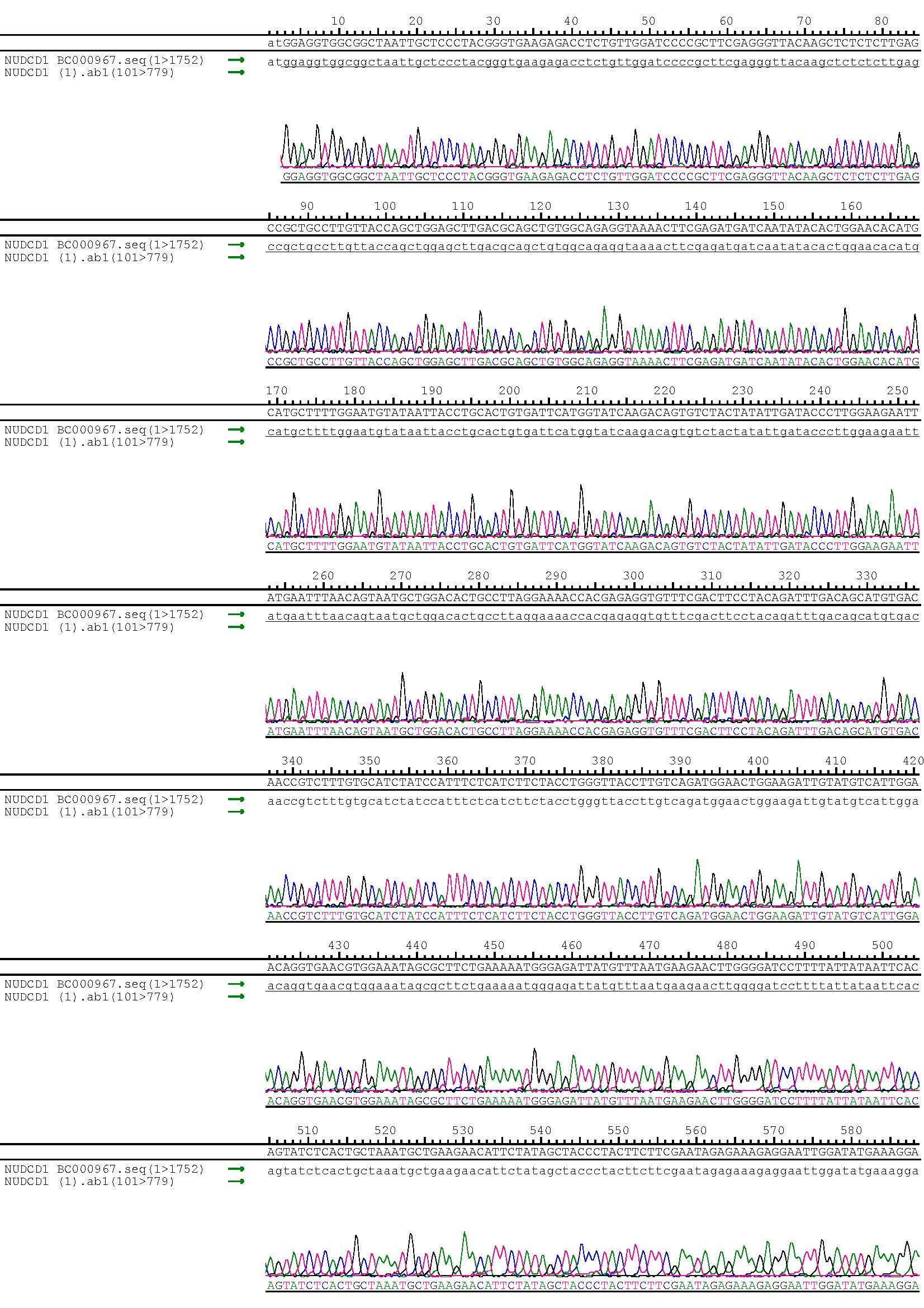

Supplement: Supplementary file 1 — Additional file 1. [file 12885_2022_10041_MOESM1_ESM.zip › supplemental figure 1-01.jpg]

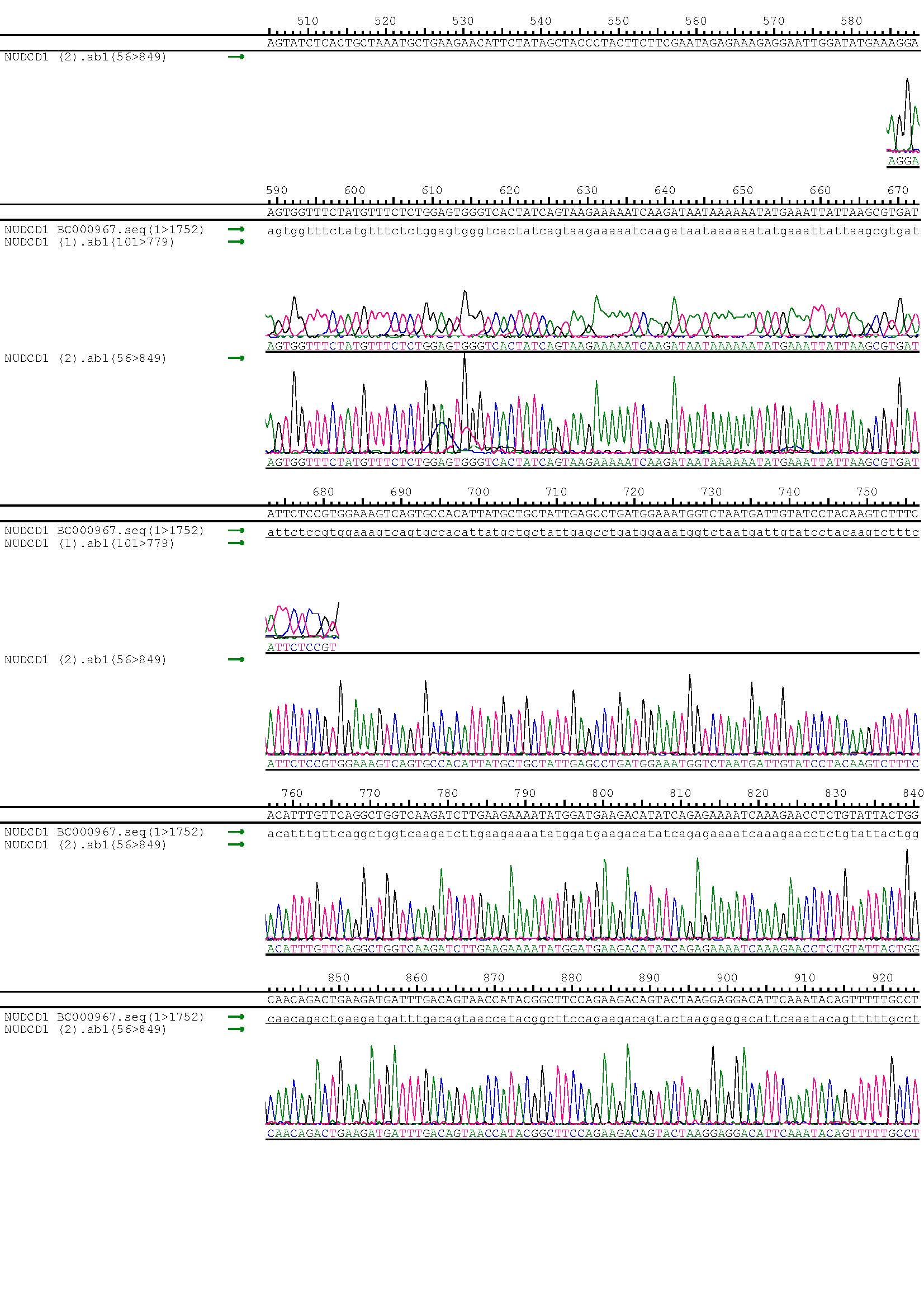

Supplement: Supplementary file 1 — Additional file 1. [file 12885_2022_10041_MOESM1_ESM.zip › supplemental figure 1-02.jpg]

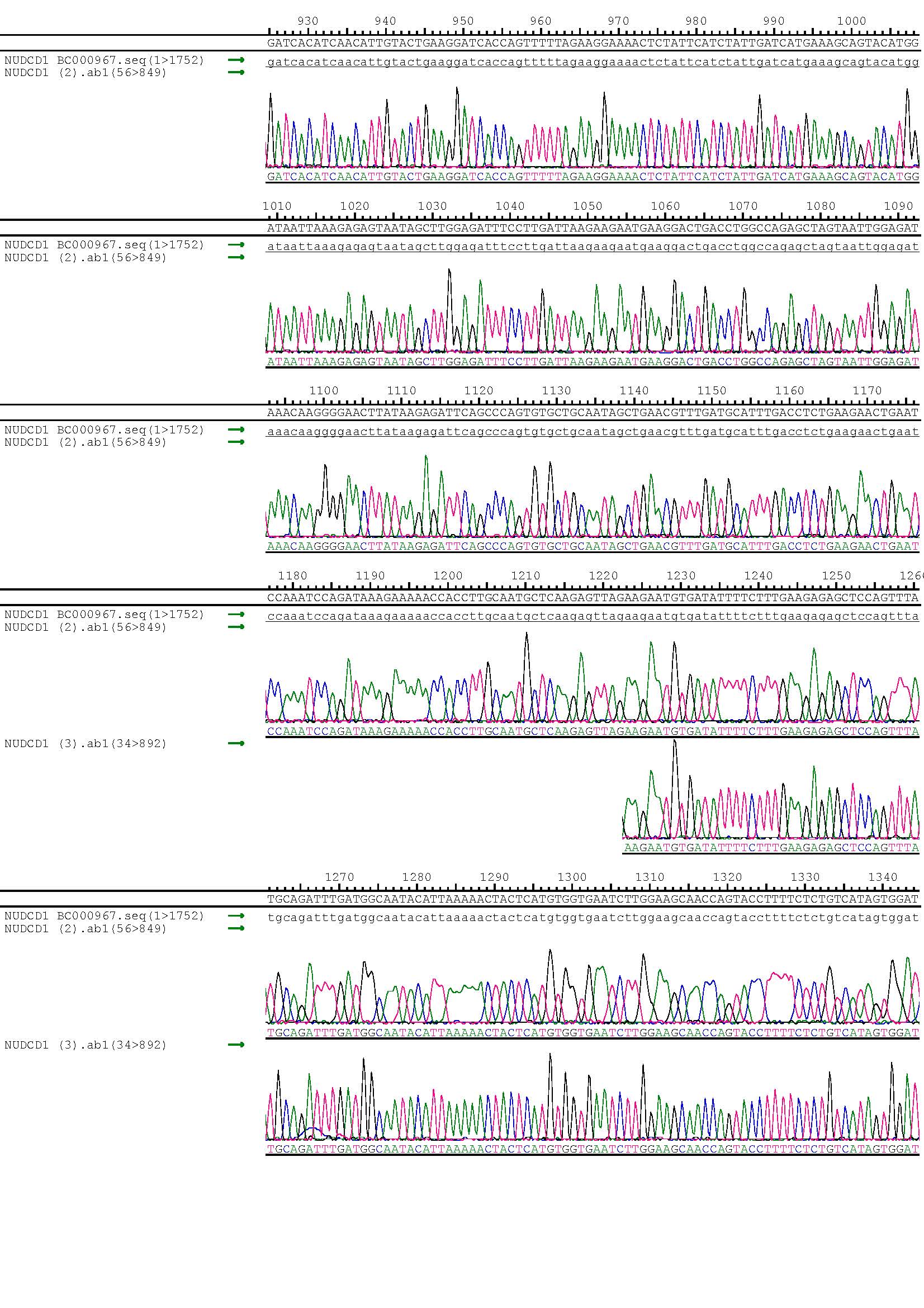

Supplement: Supplementary file 1 — Additional file 1. [file 12885_2022_10041_MOESM1_ESM.zip › supplemental figure 1-03.jpg]

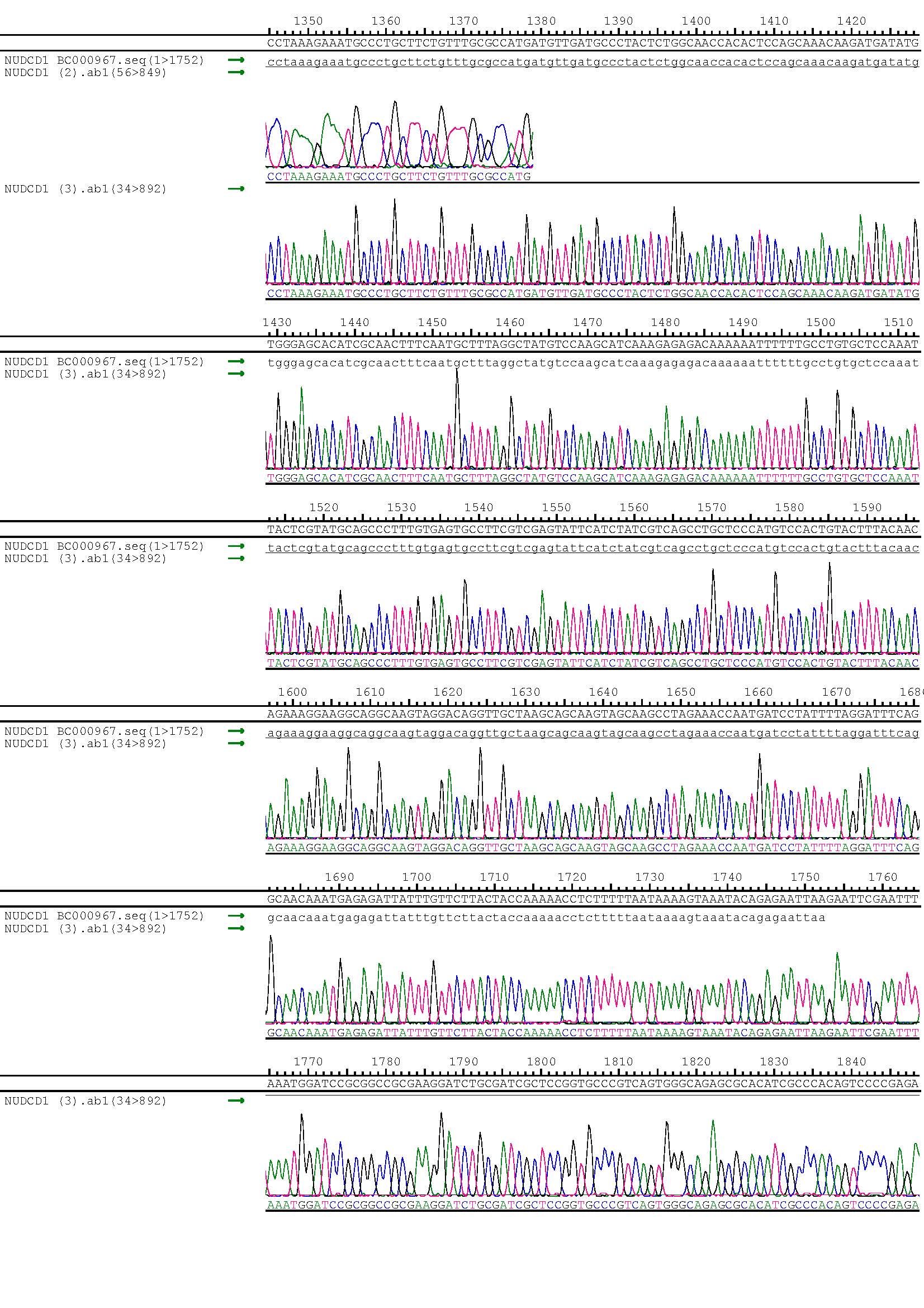

Supplement: Supplementary file 1 — Additional file 1. [file 12885_2022_10041_MOESM1_ESM.zip › supplemental figure 1-04.jpg]
